# NK Cell Subset Redistribution during the Course of Viral Infections

**DOI:** 10.3389/fimmu.2014.00390

**Published:** 2014-08-14

**Authors:** Enrico Lugli, Emanuela Marcenaro, Domenico Mavilio

**Affiliations:** ^1^Unit of Clinical and Experimental Immunology, Humanitas Clinical and Research Center, Milan, Italy; ^2^Dipartimento di Medicina Sperimentale, Centro di Eccellenza per le Ricerche Biomediche, Università degli Studi di Genova, Genova, Italy; ^3^Department of Medical Biotechnologies and Translational Medicine, University of Milan, Milan, Italy

**Keywords:** viral infection, immune escape, immune activation, innate immune response, physiophysiological interaction

## Abstract

Natural killer (NK) cells are important effectors of innate immunity that play a critical role in the control of human viral infections. Indeed, given their capability to directly recognize virally infected cells without the need of specific antigen presentation, NK cells are on the first line of defense against these invading pathogens. By establishing cellular networks with a variety of cell types such as dendritic cells, NK cells can also amplify anti-viral adaptive immune responses. In turn, viruses evolved and developed several mechanisms to evade NK cell-mediated immune activity. It has been reported that certain viral diseases, including human immunodeficiency virus-1 as well as human cytomegalovirus infections, are associated with a pathologic redistribution of NK cell subsets in the peripheral blood. In particular, it has been observed the expansion of unconventional CD56^neg^ NK cells, whose effector functions are significantly impaired as compared to that of conventional CD56^pos^ NK cells. In this review, we address the impact of these two chronic viral infections on the functional and phenotypic perturbations of human NK cell compartment.

## Introduction

In the absence of drugs that are able to eradicate and cure viral infections, the presence of efficient immune responses is key in the control and clearance of virally infected cells. Natural killer (NK) cells represent the first line of defense against viral infections, as it became clear since the first experimental evidence in the late 1980s reporting severe and recurrent herpes virus infections in a young patient with NK cell deficiency (1). The fact that NK cells do not need a prior antigen sensitization makes them ready to fight against pathogens starting from the early phases of innate immune responses through several effector functions controlled by a dynamic balance between inhibitory and activating NK cell receptors (NKRs) (2). Indeed, NK cells are able to lyse “non-self” cellular targets while sparing normal cells that express adequate levels of “self” major histocompatibility complex of class I (MHC-I) molecules. This cytolytic function is regulated by a heterogeneous family of inhibitory NKRs (iNKRs) that bind specifically to either classical or non-classical human leukocyte antigen (HLA) alleles (3). Diminution or absence of expression of HLA-I molecules on the surface of virally infected cells results in reduced engagement of iNKRs which, in turn, allow a large group of activating NKRs (aNKRs) to trigger cytotoxicity. The “on signal” exerted by aNKRs to trigger NK cell killing depends on the induced expression of putative ligands for activating receptors on virally infected target cells. The recognition of these specific ligands is required for the engagement of aNKR-mediated downstream pathways associated with the NK cell release of lytic granules (4–13).

Upon activation, NK cells also produce several chemokines such as CCL3/MIP1α, CCL4/MIP1β CCL5/RANTES, and cytokines such as interferon-γ (IFN-γ), tumor necrosis factor (TNF), and granulocyte/macrophage colony-stimulating factor (GM-CSF). These soluble factors play not only an important regulatory role in hematopoiesis and cellular activation, but are also involved in the suppression of human immunodeficiency virus-1 (HIV-1) replication through non-cytolytic mechanisms (2, 10, 14–17). NK cells are also endowed with the ability of either priming or taking part to cellular networks of interactions. In fact, it has been shown that NK cells are engaged in an active and bi-directional cross talk with autologous dendritic cells (DCs) through a process that requires both NK cell–DC cellular interactions and secretion of specific cytokines (18–25). Furthermore, monocytes/macrophages and neutrophils have been shown to regulate the recruitment and the activation of NK cells, which, in turn, can eliminate over-stimulated macrophages (26–29). It has been also reported that human neutrophils are able to establish a network with both NK cells and 6-sulfo LacNAc+ DCs (slanDC). This “*mènage à trois*” involves direct reciprocal interactions as well as positive amplification loops mediated by cell-derived cytokines with the aim of inducing IFN-γ production by NK cells (30). The final outcome of these synergic NK cell interactions is the coordination and optimization of both innate and adaptive immunity in response to inflammatory stimuli such as viral infections at tissue sites (31).

Under physiological conditions, the distribution of the surface markers CD56 and CD16 (FcγRIII) defines two subsets of CD14^neg^/CD3^neg^/CD19^neg^ NK lymphocytes: the CD56^bright^/CD16^neg-dim^ (CD56^bright^) population that accounts for ~10% of blood NK cells and the CD56^dim^/CD16^bright^ (CD56^dim^) cells that comprise for ~90% of circulating NK cells (17, 32). CD56^bright^ NK cells exert only marginal cytotoxic capacity and yet produce high amounts of cytokines like IFN-γ, TNF, and GM-CSF. Their degree of proliferation in response to activation stimuli is much higher as compared to that of CD56^dim^ NK cells. Given the pleiotropic roles of the cytokines on multiple immune and non-immune populations, CD56^bright^ NK cells have been generally referred to as regulatory NK cells. Conversely, CD56^dim^ NK cells were originally identified as the main subset endowed with cytotoxic capacity, although subsequent works indicated that they can also produce relatively high amounts of pro-inflammatory cytokines following the engagement of aNKRs (32–36). The different functional outcomes of CD56^bright^ and CD56^dim^ NK cells are associated with different repertoires of NKRs and with distinct homing capacities that are determined at the level of chemokine receptor expression on the cell surface. Indeed, CD56^dim^ NK cells preferentially migrate to inflamed peripheral tissues on the basis of their increased expression of CXCR1, CX3CR1, and ChemR23, while the CD56^bright^ subset expressing CCR7 preferentially homes to secondary lymphoid organs (37–39). Remarkably, recent data indicate that, in several pathological conditions including viral infections, CD56^dim^ NK cells may also express CCR7 *de novo* and migrate toward lymph nodes (40–42).

It is well known that viruses remarkably affect NK cell homeostasis, phenotype, and functions, thus highlighting the key roles played by NK lymphocytes in the physiopathology of chronic and inflammatory viral disorders. This review provides an updated summary of the virally induced changes of NK cell phenotype and functions and of their implications in NK cell physiology and physiopathology.

## NK Cell Responses to HIV-1

### High frequencies of CD56^neg^ NK cell subset in HIV-1 infection

Although the NK cell population is mainly composed by the two CD56^bright^ and CD56^dim^ subsets, low frequencies of a CD14^neg^/CD3^neg^/CD19^neg^/CD56^neg^/CD16^bright^ (CD56^neg^) population are also detected in healthy donors (16, 43). This unusual and rare population has been substantially ignored until mid 1990s, when it has been described that the decrement of absolute numbers of circulating NK cells during the course of HIV-1 infection is associated with expansion of an unconventional subset of CD56^neg^ NK lymphocytes (44). This report opened a new research topic in the field of NK cell biology and many groups, including ours, highlighted the great importance of CD56^neg^ NK cell in the physiopathology of HIV-1 infection. It then became evident that NK cells are remarkably affected by the deleterious effect of ongoing HIV-1 replication. Although NK cells are not productively infected by HIV-1, high and chronic levels of viremia significantly impair NK cell-mediated host immune responses, thus leading to a defective control of viral spreading and, subsequently, to disease progression. This is due, at least in part, to the defective capacities of NK cells from viremic HIV-1-infected patients to eliminate autologous HIV-1-infected CD4^pos^ T cells. Moreover, NK cells from the same individuals displayed impaired killing of cell targets either tumor-transformed or infected by opportunistic pathogens as well as weaker production of anti-viral cytokines/chemokines and defective interactions with autologous DCs (10, 17). In turn, dysfunctions in NK-DC crosstalk impair the maturation of DCs that, instead of priming an effective adaptive immune response by presenting HIV-1 antigens to T cells, contribute to disseminate the infection in secondary lymphoid organs (23). These NK cell aberrancies are a direct consequence of the HIV-1-driven expansion of the highly anergic CD56^neg^ NK cell subset. In patients with chronic or late stage HIV-1 infection and high viral loads, decreased frequencies of CD56^dim^/CD16^pos^ NK cell populations are counterbalanced by increased percentages of these dysfunctional CD56^neg^ cells expressing an aberrant repertoire of inhibitory and aNKRs. This experimental evidence clarified that, rather than an absolute decrement of total circulating NK cells (44), HIV-1 viremia is associated with a significant and pathological redistribution of NK cell subsets associated with impaired anti-viral responses (12, 16, 23, 45–53). The sequential deregulation of NK cell subset has been reported to start from the early phases of HIV-1 infection due to the presence of surface markers highly sensitive to viral replication (33, 53). In particular, it has been reported that the c-lectin-type molecule Siglec-7 (also known as p75/AIRM1), an inhibitory receptor constitutively expressed on all NK cells, is the first marker to be down-regulated during the early phases of HIV-1 infection before the loss of CD56. Siglec-7 down-modulation is preserved throughout the course of the infection and depends on the level of viral replication. Indeed, the small cohort of individuals that do not progress toward AIDS (i.e., the long-term non-progressors) and who naturally display low or undetectable HIV-1 viremia keep a normal distribution of NK cell subsets as identified by the expression of Siglec-7 and CD56. Since all these NK cell phenotypic and functional abnormalities are reversible following a successfully suppression of viral replication, the pathological redistribution of NK cell subsets can also be used to monitor the effectiveness of antiretroviral therapy (ART) (17).

Finally, we recently reported that the NK cell modulation of Siglec-7 in HIV-1 infection is directly involved in HIV-1 pathogenesis (54). In fact, chronic high levels of viral replication lead to a decreased surface expression of Siglec-7 on NK cells counterbalanced by increased levels of soluble Siglec-7 detected in the plasma of viremic HIV-1-infected patients. This soluble form of Siglec-7 is able to directly bind the glycoprotein 120 expressed on HIV-1 envelope and facilitates the infection of Siglec-7^neg^/CD4^pos^ T cells. In contrast, high levels of HIV-1 viremia do not alter the constitutive expression of Siglec-7 in monocytes and macrophages, whose susceptibility to HIV-1 infection is enhanced by the direct interaction between the virus and this lectin-type receptor (33, 54). These data suggest that, similar to other members of Siglec family (55–57), both membrane-bound and soluble Siglec-7 greatly increase the susceptibility of CD4^pos^ cell targets expressing CCR5 or CXCR4 to be infected by HIV-I (54).

### Origin of the CD56^neg^ NK cell subset

Natural killer cells resulted not to be directly infected by HIV-1 (45) and, therefore, it is unlikely that the expansion of CD56^neg^ NK cell is HIV-1 specific. Indeed, although the origin of CD56^neg^ cells is still being debated, it later became clear that high frequencies of this pathological subset are associated with the presence of chronic and systemic inflammation, which is a hallmark of chronic HIV-1 infection (10, 12). Several studies then investigated other human disorders characterized by high levels of systemic immune activation and reported similar increased percentages of circulating CD56^neg^ NK cells. Among these diseases, there are hepatitis C virus (HCV) (58, 59), human cytomegalovirus (HCMV) (60), hantavirus (61), treponema pallidum (62) infections, post-transplant lymphoproliferative malignancies driven by Epstein–Barr virus (EBV), (63) and autoimmune disorders such as myasthenia gravis (64) and dermatomyositis (65). Expansion of CD56^neg^ NK cells has been described both in HCV as well as in HCV–HIV co-infected patients (59). However, the increase of this aberrant subset is much more contained in mono-infection by HCV compared to HCV–HIV co-infection. Therefore, additional studies are required to clarify whether the accumulation of CD56^neg^ NK cells is a hallmark of chronic HCV infection. Finally, a significant proportion of CD56^neg^ cells have also been found in umbilical cord blood and in healthy infants and are characterized by impaired anti-viral activities (66–69).

The fact that high frequencies of CD56^neg^ NK cells are found in so many different either pathological or physiological conditions underline that their ontogenesis relies on mechanism(s) associated with activation of the immune system and not with a specific viral infection or inflammatory disorder. Moreover, in all the above-mentioned disorders as well as in umbilical cord blood, CD56^neg^ cells share the same phenotypic and functional features: (i) low expression of natural cytotoxity receptors (NCRs) and Siglec-7; (ii) reduced cytolytic potential; (iii) decreased production of anti-viral cytokines and chemokines. In regard to their ontogeny, it has been first postulated that this “anergic” CD56^neg^ cells could arise from a failure of NK cell development and/or from inadequate cell stimulation. This theory was mainly supported by experimental evidence showing that the incubation *in vitro* of CD56^neg^ cells with IL-2, IL-12, and IL-15 induces cell proliferation and restores the classical distribution of CD56 and repertoire of NKRs (44, 59, 67). We have to point out though that therapies with anti-viral drugs (in case of HIV-1 and HCV infections) or with immunosuppressants (for myasthenia gravis and dermatomyositis) restored physiological NK cell phenotype and functions (16, 59, 64, 65).

Unfortunately, there are no reports showing that an *in vitro* setting could reproduce the expansion of CD56^neg^ cells and this hampered our capacity to disclose the mechanistic insights highlighting this phenomenon. Our current knowledge of NK cell ontogenesis states that CD16^neg^ immature NK (iNK) cells expressing low levels of CD56 and NCRs precede CD56^bright^ NK cells in development (43, 70). Although sharing these phenotypic features with iNKT, CD56^neg^ NK cells also express many NK cell-specific receptors that are not found on iNK cells, including KIRs, CD94/NKG2A, NKG2D and CD16 (10, 43). iNK cells produce high amounts of GM-CSF but not other inflammatory cytokines following phorbol myristate acetate (PMA)/ionomycin stimulation, and they are not even able to kill target cells nor to produce cytokines (70). Moreover, iNK cells develop into CD56^bright^ NK cells after stimulation with IL-15, thus suggesting they are precursors of these cells *in vivo* (70). On the contrary, CD56^neg^ NK cells retain inflammatory cytokine production and killing capacity, albeit at impaired level compared to conventional CD56^pos^ NK cells (10, 17). Finally, CD56^neg^ cells but not iNK cells have been recently shown to express CD57 (71), a marker of terminal cell differentiation (72). Taken together, these experimental findings strongly suggest that CD56^neg^ NK cells do not derive from iNK cells.

An alternative hypothesis proposed that CD56^neg^ NK cells are mature lymphocytes that recently engaged target cells. Under normal circumstances, NK cells are capable of killing multiple target cells, thus resulting in a reduced, but never complete, loss of perforin and granzyme B (73). In HIV-1-infected patients, decreased granzyme B and perforin expression and increased surface expression of CD107a in the absence of *ex vivo* stimulation suggests that CD56^neg^ NK cells engage target cells *in vivo*. Authors also argued that this hypothesis is further supported by the increased expression of CD95 on CD56^neg^ cell subset, thus indicating a more pronounced activation compared to their CD56^dim^ cell counterpart (71). However, the down-regulation of CD56 as a consequence of recent activation is still awaiting confirmation by additional studies. Since the transcriptome of CD56^neg^ NK cells has been found to be more similar to myeloid cells than to traditional CD56^dim^ NK cells (74), CD7, a surface protein expressed on thymocytes and mature T cells, has been proposed as an additional informative marker for their identification. In this regard, CD56^neg^ NK cells have been reported to be a mixed population of CD7^pos^ true NK cells and CD7^neg^ myeloid cells present at a low frequency in healthy donors and expanded in HIV-1 viremic subjects (71).

## NK Cell Responses to Human Cytomegalovirus

Similar to what it has been observed in humans (1), mice infected with murine cytomegalovirus (MCMV) and depleted of NK cells were unable to control infection and displayed disseminated MCMV in the lungs and in the liver (75). The anti-viral NK cell responses are particularly relevant when viruses exploit mechanisms of immune evasion, leading to the down-regulation of MHC-I molecules and thus escape from the CD8^pos^ T cell-mediated cytolytic activity. Multiple HCMV-derived proteins have been demonstrated to interfere with the transport or the expression of MHC-I on the cell surface (76). On the other hand, HCMV-infected cells up-regulate ligands for the aNKR NKG2D, including MICA, MICB, and UL-16 binding proteins (ULBPs), thereby facilitating NK cell activation (77–79). On average, 50–80% of the human population in Western countries is HCMV-infected, but the virus does not harm the health of immune-competent individuals, unless in specific situations such as maternal HCMV reactivation or primary infection during pregnancy. Instead, immune-compromised individuals, such as those infected with HIV-1 or those who received bone marrow transplantation (BMT), are particularly susceptible to CMV reactivation (80). Following BMT, NK cells recover faster than CD8^pos^ T cells (2–3 weeks vs. 4–6 weeks, depending on the type of transplantation) and possibly mediate a first line of protection against viral dissemination (81). Different groups have recently shown that patients experiencing HCMV reactivation following BMT display highly mature KIR^pos^, NKG2A^neg^, NKG2C^pos^, and CD57^pos^ NK cells that are not found in uninfected recipients (82–84). A similar NK cell phenotype is observed following acute HCMV infection in healthy individuals (72). Interestingly, modification of the NK cell surface phenotype did not change with resolution of the infection (83), thus suggesting a stable imprinting in the NK cell maturation stage. Similar to what it has been observed in HCV and HIV-1 infections (33, 43), HCMV-reactivations in patients undergoing umbilical cord blood transplantation induce the expansion of the CD56^neg^/CD16^pos^/Siglec-7^neg^ NK cell subset (60). The expansion of anergic CD56^neg^ NK cells following HCMV reactivation likely occurs when T cell immunity is impaired and supports the hypothesis that HCMV has a role in immune-senescence (85, 86).

Multiple components of the immune system are thus engaged to protect the host from reactivating CMV replication. The sole NK cell response is likely not sufficient in this regard and must act in concert with recovering CMV-specific CD8^pos^ T cells with the support of CMV-specific CD4^pos^ T cells. This is well demonstrated in individuals infected with HIV, where the loss of antigen-specific CD4^pos^ T cells causes increased susceptibility to opportunistic infections, including CMV reactivation (87). These data altogether suggest that NK cells could be exploited together with CD8^pos^ T cells in the therapeutic treatment of CMV reactivation.

### HCMV and memory NK cells

Studies in mice led to the demonstration that a population of “memory” NK cells develop following acute infection with MCMV. In particular, it has been reported that NK cells expressing the activating Ly49H receptor undergo a clonal-like expansion upon recognition of the MCMV-encoded m157 antigen and generate long-lived memory NK cells (88). Similarly in humans, it has been recently demonstrated that NKG2C^pos^ NK cells remarkably increase in frequency following HCMV infection or reactivation and can persist for years (60, 72, 89–92). Such expanded NKG2C^pos^ NK cells may exert an efficient anti-viral activity by producing higher amounts of cytokines, in particular IFN-γ, hence suggesting that “memory-like” NK cell responses may occur in humans as well. However, NKG2C cannot be considered as a univocal marker of “memory-like” NK cells. Indeed, recent reports suggest that also other NKRs that are preferentially found in terminally differentiated NK cells, including activating KIRs or CD57, are up-regulated following HCMV reactivation. In this context, a number of recent studies suggest that the presence of activating KIRs correlates with protection against viral infections (84, 93).

Despite “memory-like” NK cells mostly display a CD56^dim^/CD16^pos^ phenotype (60, 72), they have been shown to share some phenotypic traits with CD56^neg^/CD16^pos^ NK cells as well. These similar traits between “memory-like” and CD56^neg^/CD16^pos^ NK cells are not supported by functional features, as the former are expected to display increased functional capacity while the latter are well known to be impaired in a number of effector functions. Further studies are needed to confirm if the KIR^pos^/NKG2A^neg^/NKG2C^pos^ CD57^pos^ NK cells represent the human memory NK cell counterpart and to test whether they share any of the CD56^neg^/CD16^pos^ NK cell properties. Importantly, it would be interesting to determine whether continuous stimulation of NK cells by persistent, but undetectable viral replication, in infected individuals plays a role in maintaining these mature NK cell phenotypes.

## Concluding Remarks

Viruses employ several strategies to escape from NK cell-mediated clearance of virally infected cells or secretion of anti-viral cytokines. In particular, it became clear that viruses are able to affect the functional status and the homeostasis of NK cells through the modulation/engagement of several surface receptors and through the expansion of unconventional NK cell subsets. Although many aspects of NK cell physiology have been disclosed by taking lessons from the physiopathology of viral infections, several fundamental questions still remain to be answered. First of all, the origin and the expansion of the CD56^neg^ NK cells subset that represents the largest fraction of total NK cells in late stages of HIV-1 infection and that highly contributes to the lack of viral control and to disease progression. Disclosing the mechanisms underlying the high frequencies of this highly anergic NK cell population is key to better understand not only NK cell development but also to make it possible the manipulation of these cells *in vitro* in order to improve their anti-viral potential and provide a better control of viral replication and spreading.

## Conflict of Interest Statement

The Review Editor Andrea De Maria declares that, despite being affiliated to the same institution as authors Emanuela Marcenaro and Domenico Mavilio, the review process was handled objectively and no conflict of interest exists. The authors declare that the research was conducted in the absence of any commercial or financial relationships that could be construed as a potential conflict of interest.
